# Overcoming barriers to evidence-based patient blood management: a restricted review

**DOI:** 10.1186/s13012-020-0965-4

**Published:** 2020-01-17

**Authors:** Alana Delaforce, Jed Duff, Judy Munday, Janet Hardy

**Affiliations:** 1 The University of Newcastle, School of Nursing and Midwifery, University Drive, Callaghan, NSW 2302 Australia; 2 School of Nursing/Institute for Health and Biomedical Innovation, Queensland University of Technology, Victoria Park Rd, Kelvin Grove, QLD 4059 Australia; 3 Faculty of Health and Sports Sciences, The University of Agder, Grimstad, Norway; 4 Mater Health Services, Level 6, Duncombe Building, Raymond Terrace, QLD 4101 Australia

**Keywords:** Patient blood management, Consolidated Framework for Implementation Research, Expert Recommendations for Implementing Change, Barrier Mapping, Implementation Strategies

## Abstract

**Background:**

Blood transfusions are associated with a range of adverse patient outcomes, including coagulopathy, immunomodulation and haemolysis, which increase the risk of morbidity and mortality. Consideration of these risks and potential benefits are necessary when deciding to transfuse. Patient blood management (PBM) guidelines exist to assist in clinical decision-making, but they are underutilised. Exploration of barriers to the implementation and utilisation of the PBM guidelines is required. This study aimed to identify common barriers and implementation strategies used to implement PBM guidelines, with a comparison against current expert opinion.

**Methods:**

A restricted review approach was used to identify the barriers to PBM guideline implementation as reported by health professionals and to review which implementation strategies have been used. Searches were undertaken in MEDLINE/PubMed, CINAHL, Embase, Scopus and the Cochrane library. The Consolidated Framework for Implementation Research (CFIR) was used to code barriers. The Expert Recommendations for Implementing Change (ERIC) tool was used to code implementation strategies, and subsequently, develop recommendations based on expert opinion.

**Results:**

We identified 14 studies suitable for inclusion. There was a cluster of barriers commonly reported: access to knowledge and information (*n* = 7), knowledge and beliefs about the intervention ( = 7) and tension for change (*n* = 6). Implementation strategies used varied widely (*n* = 25). Only one study reported the use of an implementation theory, model or framework. Most studies (*n* = 11) had at least 50% agreement with the ERIC recommendations.

**Conclusions:**

There are common barriers experienced by health professionals when trying to implement PBM guidelines. There is currently no conclusive evidence to suggest which implementation strategies are most effective. Further research using validated implementation approaches and improved reporting is required.

Contributions to the literature

- Our review is the first to provide synthesised evidence regarding the barriers to patient blood management (PBM) guidelines.
- Our review reports implementation strategies used, then classifies and compares them against the Expert Recommendations for Implementing Change (ERIC) tool.
- Our review confirms that the reporting of implementation methods and implementation strategies used to enhance the uptake of PBM guidelines is currently limited and makes recommendations on how to improve the reporting of future studies.

## Background

Blood transfusions carry significant risks to patient safety and should be used sparingly [1, 2]. Such risks include immunomodulation (where the patient acquires new antibodies, making it harder to locate compatible blood products), coagulopathies (increased risk of venous thromboembolism and pulmonary embolism), haemolysis (red cell destruction) and adverse reactions (including transfusion-associated circulatory overload and lung injury) [1–3]. Given the risk of morbidity and mortality associated with blood transfusions, it is crucial that patients only receive blood transfusions where the potential benefit outweighs these risks.

Globally, patient blood management (PBM) guidelines have been developed to provide clarity and support to clinicians when considering transfusion [4–7]. The guidelines consider three key principles, or “pillars” when making recommendations: the maximisation of a patient’s red cell mass before invasive procedures, the minimisation of intraoperative blood loss and that patients are supported to tolerate anaemia rather than receive a blood transfusion [8, 9]. When implemented effectively, the guidelines can have a significant impact on improved patient care [8, 10, 11]. A systematic review published in 2018 found that implementation of a multimodal PBM program (using the three pillars) resulted in a 39% reduction in transfusion rates, in addition to statistically significant reductions in hospital length of stay and an overall reduction of 11% in mortality rates [8].

Many implementation strategies that support the implementation of PBM guidelines have been developed and utilised, but it is not clear which are the most effective [10–24]. Some examples of implementation strategies used to improve the uptake of PBM guidelines include using local consensus processes, audit and feedback, providing education and identifying and preparing champions [10–23]. A systematic review by Tinmouth and colleagues found the use of behavioural implementation strategies to be effective at reducing blood product utilisation, but due to heterogeneity across studies, they could not make specific recommendations [25]. These difficulties are not unique to PBM guideline implementation, and much research has been undertaken to help advance the language, processes used and reporting of experiences to help provide clarity and direction to improve the translation of evidence to practice [26, 27].

There are several frameworks available in the literature that health professionals can use to identify barriers, guide intervention selection and support the implementation process [26, 28–30], such as the Consolidated Framework for Implementation Research(CFIR) that is utilised in this review [29]. The CFIR was developed to provide a unified taxonomy of existing frameworks and was the result of a systematic review of 19 existing frameworks [29]. The CFIR comprises five domains, and 39 theoretical constructs thought to influence implementation [31, 32]. The five domains include the intervention, the inner setting, outer setting, individuals involved and the process by which implementation is accomplished [29]. The CFIR also provides a comprehensive data dictionary that specifies what each construct means to assist with correct coding [29]. On its own, the CFIR is useful, but historically, it was not easily mapped to other tools to assist with implementation strategy selection, following barrier identification. A recently developed tool: Expert Recommendations for Implementing Change (ERIC) helps to address this limitation [33]. The ERIC implementation strategy selection tool comprises 73 strategies to enhance implementation [34]. The implementation strategies were compiled by 71 experts over three Delphi rounds in an attempt to gain consensus on what implementation strategies positively influence implementation [34]. The definitions of the implementation strategies are also outlined in a data dictionary to help guide correct classification [34]. The ERIC tool allows the user to select the relevant local barriers (as classified by the CFIR) and generate a list of implementation strategies that, according to expert opinion, should be effective in addressing them [33]. The ERIC tool is one of the many options that can be used to understand implementation problems. To date, the reported use of such frameworks and tools to guide implementation of patient blood management guidelines has been limited [33].

This review will examine implementation strategies used to address barriers to implementing patient blood management guidelines. Specifically, it aims to highlight the barriers identified by health professionals and any implementation strategies used. These are then compared against current expert opinion, based on the assumption that better selection of implementation strategies leads to improved translation of evidence into practice.

## Methods

### Approach

**Fig. 1:**
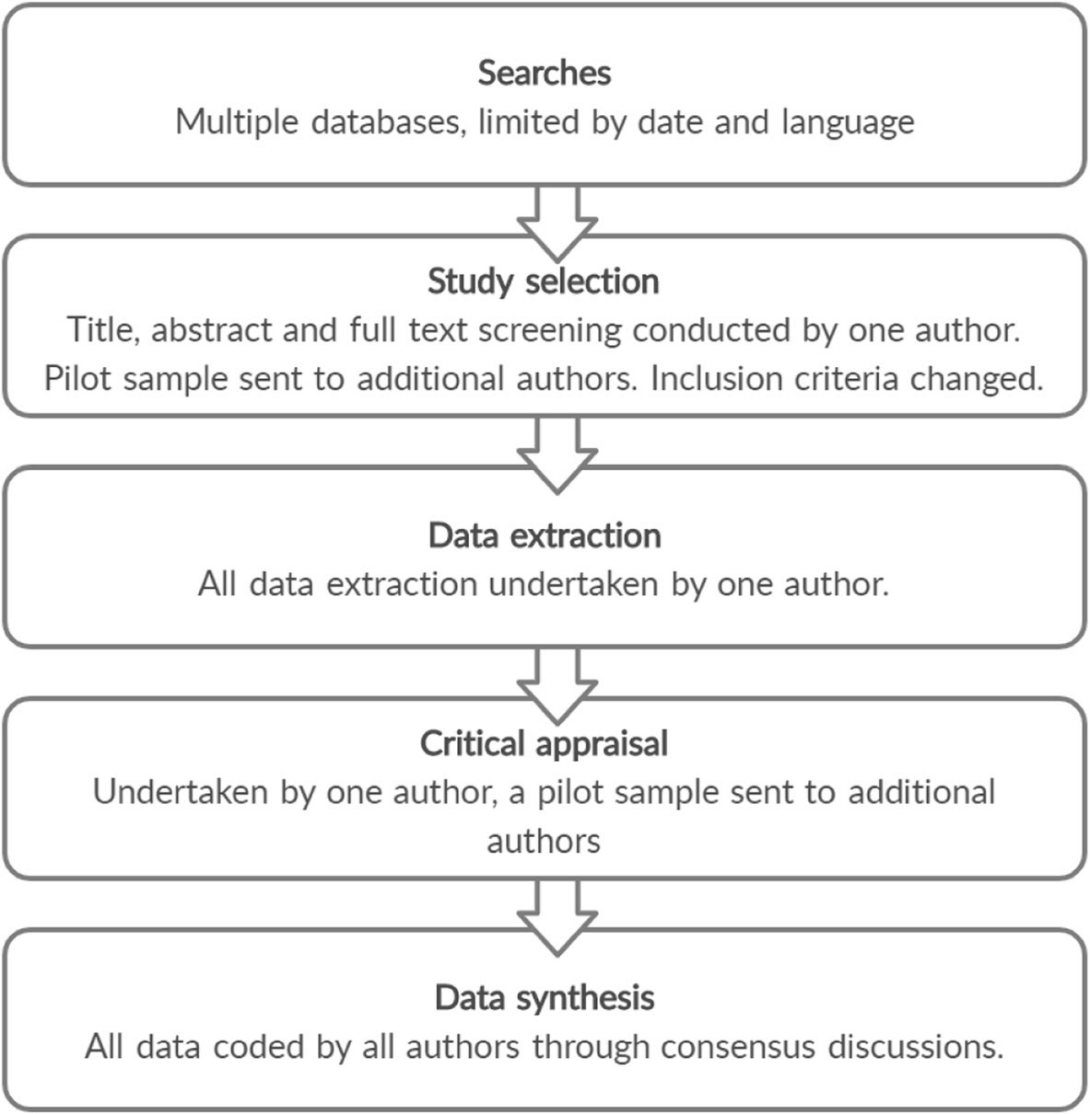
Process of restricted review

We utilised a newly described restricted systematic review approach, as proposed by Plüddeman and colleagues [35]. In the context of limited resources, the restricted review approach uses a flexible framework to select the level of rigour at each phase of the review [35]. The level of rigour is determined by the level of input from the team. For example, in a traditional review, two members are responsible for the title and abstract screening, whereas, in a restricted review, these may be undertaken by one author only [35]. In keeping with this method, we used pilot sampling during screening, study selection and quality assessment phases. An overview of the process is shown in Fig. 1.

### Searches

We searched for publications that had the word “blood” and “implement*”, “manage*” or “guideline*” in the title and excluded irrelevant terms (such as sugar, glucose, pressure and cholesterol). We included published literature only, in the Cumulative Index for Nursing and Allied Health Literature (CINAHL), Embase (Ovid interface, 1948 onwards), MEDLINE (Ovid interface, 1948 onwards), Scopus and Cochrane library database. The initial search was undertaken in March 2018 and repeated in June 2019 to confirm there were no new relevant articles. We also hand-searched further articles by scanning references lists of full-text articles. After the removal of duplicates, one author completed the title and abstract screening in Covidence™ (See Fig. 1. ).

### Study selection and data extraction

**Fig. 2:**
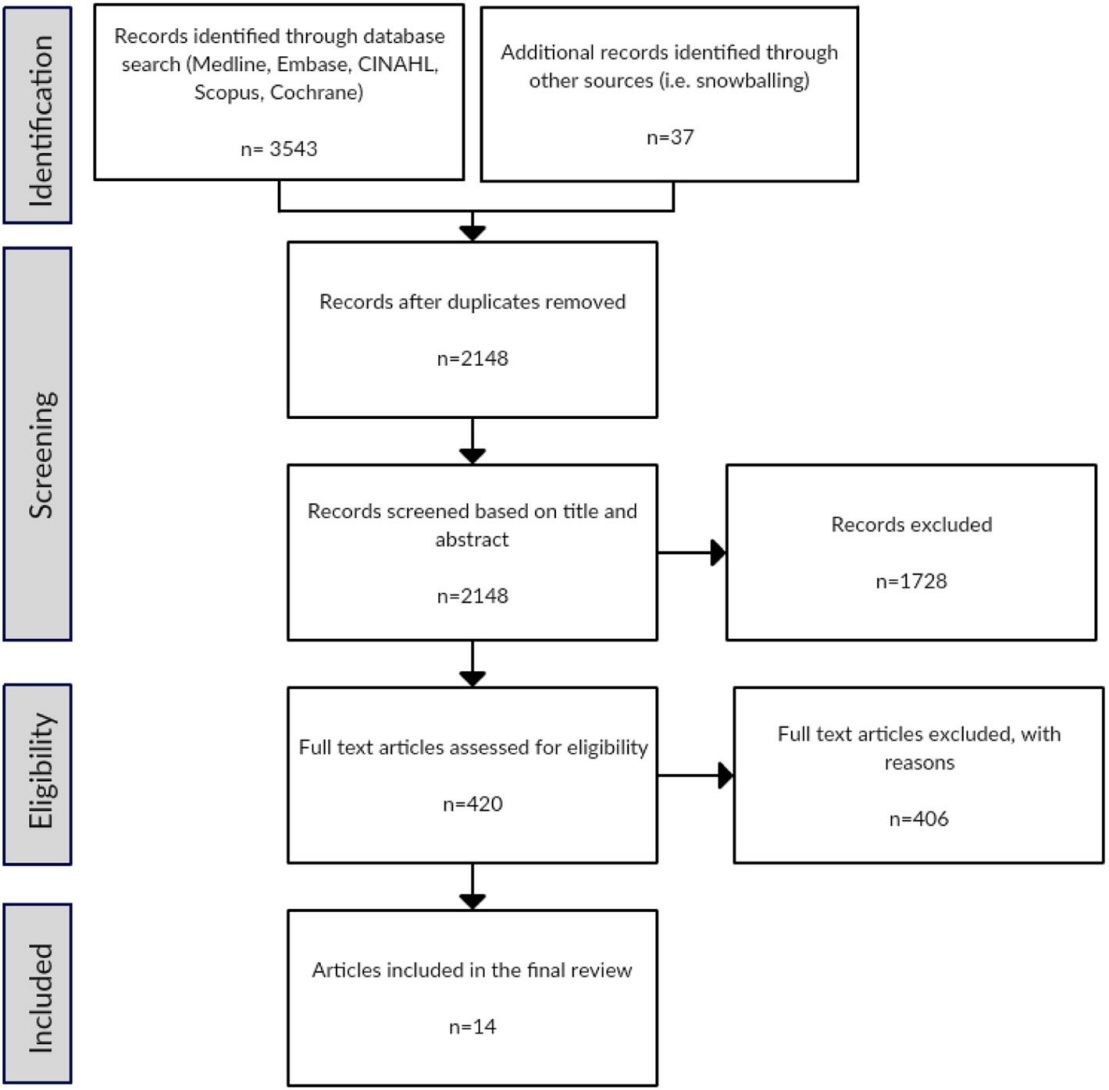
Prisma flow diagram

Articles were eligible for inclusion if they were a primary research study of any design comparing PBM implementation strategies with usual or standard care, had identified barriers to implementing PBM guidelines and had been written in English and published between 1999 and 2019. This date range was chosen as the landmark study highlighting the risks associated with blood transfusions was published in 1999 [36]. We defined barriers as existing impediments to the uptake of the PBM guidelines. During the initial full-text screening, the second and third authors were blinded to the first author’s decision. Resolution by consensus occurred where there was disagreement at this point, and the pilot screening process revealed the need for tighter inclusion criteria (finalised as per above). The amended criteria were then applied to all remaining articles. Post hoc adjustments to inclusion criteria are acceptable in restricted reviews such as this during pilot screening, when additional authors are reviewing full texts, and consensus discussions are taking place [35, 37, 38]. The PRISMA flow diagram [39] included details of the characteristics of excluded studies (Fig. 2). Data were extracted by one author (AD) using an online data extraction form. We collected demographic data, including geographic location, patient population, study design, research methods, barriers and implementation strategies used (Table 1). We also collected the reduction in red cell utilisation but did not undertake a full analysis as this has been addressed in a previous systematic review by Tinmouth and colleagues [25].

**Table 1:** Included study demographics

Authors	TItalicheory/model	Location	Study design	MMAT QA score (%)	Patient population	Outcome
Abbett et al. (2015) [12]	Nil	North America	Retrospective observational	75	Hospital-wide	Reduction 14.3% excess transfusion
Albinarrate et al (2015) [13]	Nil	Europe	Retrospective observational	100	Perioperative adults	Transfusion reduction hip surgery 17%; knee surgery 21.6%
Ansari and Szallasi (2012) [14]	Nil	North America	Observational, prospective audit	75	Adults	Transfusion reduction 6%
Brevig et al. (2009) [15]	QI	North America	Prospective interventional	100	Perioperative	Transfusion reduction 25%
Cohn et al. (2014) [16]	Nil	North America	Retrospective audit	50	Adults, paediatric and neonatal	RBC transfusion/1000 patient days reduced 67%
Garrioch et al. (2004) [17]	Nil	Europe	Prospective interventional	75	Hospital-wide	Number patients transfused 0.9% reduction
Kumar et al. (2011) [18]	Nil	North America	Qualitative	75	Hospital-wide	Units per 1000 patient days – unclear result
Mallett et al (2001) [19]	Nil	Europe	Prospective audit (mixed methods)	75	Perioperative	Transfusion reduction 43%
Oliver et al. (2014) [20]	Nil	Europe	Quasi-experimental (before and after)	100	Hospital-wide	43% reduction units per patient discharged
Pearse et al. (2015) [21]	Nil	Australia	Retrospective observational	100	Perioperative	Transfusion reduction 15.1%
Rineau et al. (2016) [22]	Nil	Europe	Prospective interventional	75	Perioperative	Transfusion reduction 10%
Szpila et al. (2015) [23]	Nil	North America	Quasi-experimental (before and after)	100	Critical care	Units per patient 1.2 to 0.7 (42% reduction)
Whitney et al. (2013) [10]	Nil	North America	Quasi-experimental (before and after)	100	Perioperative neonatal and paediatric	66% reduction odds ratio reduction for transfusion
Zuckerberg et al. (2015) [11]	Nil	North America	Retrospective observational	100	Perioperative	Transfusion reduction 14.3%

Legend 1: *Nil* = no theory or model used, *QI* = quality improvement named as method used

### Quality assessment

The Mixed Methods Appraisal Tool (MMAT) was used for quality assessment as this facilitates rapid concurrent quality assessment across qualitative, quantitative and mixed methods studies [40]. The MMAT tool has two screening questions and four criteria (three for mixed methods studies) that the user nominates as being present or absent in each article [40]. For each criterion present, a score of 25% is awarded to the study. If all four criteria are met, then a score of 100% is assigned. Criteria are designed to gauge the reliability of the information and assess sample sizes, measurements used and whether there was a complete dataset [40].

### Data synthesis and presentation

Data extracted were exported into an Excel™ spreadsheet and collated into tables to facilitate the coding of barriers, implementation strategies and agreement with the ERIC tool recommendations [33]. The CFIR framework supported the classification and coding of barriers [29], and the ERIC classification tool supported implementation strategy coding [33]. Both associated data dictionaries provided coding guidance [29, 33, 34]. Multiple coding and classification of individual statements occurred where necessary. Consensus discussions between all three reviewers facilitated the full coding agreement.

Details of the implementation strategies used in each study to address identified barriers and the agreement with the ERIC tool for each paper are provided in Table 2. The barriers from each study were entered into the ERIC tool, which provides a list of recommended implementation strategies based on the barrier selection made [33]. The ERIC tool provides categories for recommendations from weak, moderate, and strong. Strong recommendations are those with over 50% expert consensus that the implementation strategy is appropriate for a given barrier, and moderate are those with a 20 to 49% consensus [33]. Agreement with the ERIC recommendations was calculated based on overall barriers present and whether or not a moderate or strong recommendation for each implementation strategy used was evident. Table 3 provides details of all the barriers, the ERIC recommended implementation strategies and highlights in italic text which recommended strategies were used to address specific barriers.

**Table 2:** Barriers, implementation strategies and ERIC agreement

Authors	CFIR construct barrier	ERIC classified implementation strategies	Strong or moderate ERIC recommendation (%)
Abbett et al. (2015) [12]	Access to knowledge and information, knowledge and beliefs about the intervention, evidence strength and quality, structural characteristics, culture	Alter incentive/allowance structures, audit and provide feedback, conduct educational meetings, involve executive boards, remind clinicians	80
Albinarrate et al. (2015) [13]	Access to knowledge and information	Conduct local consensus discussions, develop educational materials.	50
Ansari and Szallasi (2012) [14]	Knowledge and beliefs about the intervention, tension for change	Remind clinicians	0
Brevig et al. (2009) [15]	Culture, knowledge and beliefs about the intervention, tension for change	Capture and share local knowledge, remind clinicians, conduct educational meetings, identify and prepare champions, develop a formal implementation blueprint, develop educational materials, Audit and provide feedback	86
Cohn et al. (2014 )[16]	Evidence strength and quality, knowledge and beliefs about the intervention, access to knowledge and information.	Distribute educational materials, develop and implement tools for quality monitoring	50
Garrioch et al. (2004) [17]	Tension for change, structural characteristics	Conduct educational meetings, conduct local consensus discussions, use mass media, develop educational materials	50
Kumar et al. (2011) [18]	Structural characteristics, access to knowledge and information, available resources, tension for change, engagement, complexity.	Conduct educational meetings, develop and organize quality monitoring systems, capture and share local knowledge, conduct local consensus discussions, Intervene with patients/consumers to enhance uptake and adherence, involve executive boards	83
Mallett et al. (2001) [19]	Knowledge and beliefs about the intervention	Conduct educational meetings, facilitate relay of clinical data to providers, promote adaptability, develop and implement tools for quality monitoring, mandate change	20
Oliver et al. (2014) [20]	Evidence strength and quality, knowledge and beliefs about the intervention, culture, peer pressure, relative advantage.	Audit and provide feedback, start a dissemination organization, develop educational materials, use data experts, conduct local consensus discussions, Conduct educational outreach visits, Involve executive boards	86
Pearse et al. (2015) [21]	Access to knowledge and information, tension for change.	Develop educational materials, conduct educational meetings, conduct ongoing training, provide ongoing consultation, facilitate relay of clinical data to providers, develop and implement tools for quality monitoring	83
Rineau et al. (2016) [22]	Access to knowledge and information.	Distribute educational materials, remind clinicians	50
Szpila et al. (2015) [23]	Knowledge and beliefs about the intervention, culture	Conduct educational meetings, audit and provide feedback, obtain formal commitments, conduct local consensus discussions	50
Whitney et al. (2013) [10]	Access to knowledge and information, tension for change	Create a learning collaborative, conduct local consensus discussions, develop educational materials, audit and provide feedback, facilitate relay of clinical data to providers, develop and implement tools for quality monitoring	83
Zuckerberg et al. (2015) [11]	Structural characteristics	Conduct educational outreach visits, audit and provide feedback, conduct educational meetings, remind clinicians, develop and implement tools for quality monitoring	0

**Table 3:** Barriers and ERIC moderate or strong recommendations

CFIR construct	ERIC strong or moderate recommendations		
Access to knowledge and information [10, 12, 13, 16, 18, 21, 22]	**Conduct educational meetings** **[**12**,** 18**,** 21**]** **Develop educational materials** **[**10**,** 13**,** 21**]** **Distribute educational materials** **[**16**,** 22**]** **Create a learning collaborative** **[**10**]** **Conduct ongoing training** **[**21**]** **Capture and share local knowledge** **[**18**]** Conduct educational outreach visits Identify and prepare champions Provide local technical assistance Shadow other experts	Structural characteristics [11, 12, 17, 18]	**Capture and share local knowledge** **[**18**]** Assess for readiness and identify barriers and facilitators Change physical structure and equipment Identify and prepare champions Conduct small cyclical tests of change Build a coalition Identify early adopters Promote adaptability Promote network weaving
Knowledge and beliefs about the intervention [12, 14–16, 19, 20, 23]	**Conduct educational meetings** **[**12**,** 15**,** 18**,** 19**,** 23**]** **Identify and prepare champions** **[**15**]** **Develop educational materials** **[**15**,** 20**]** **Conduct educational outreach visits** **[**20**]** **Capture and share local knowledge** **[**15**,** 18**]** Conduct a local needs assessment Assess for readiness and identify barriers and facilitators Facilitation Identify early adopters Increase demand Stage implementation scale-up Inform local opinion leaders	Evidence strength and quality [12, 16, 20]	**Conduct educational meetings** **[**12**]** **Conduct local consensus discussions** **[**20**]** **Conduct educational outreach visits** **[**20**]** **Distribute educational materials** **[**16**]** **Develop educational materials** **[**20**]** Capture and share local knowledge Develop academic partnerships Identify early adopters Identify and prepare champions Inform local opinion leaders
Culture [12, 15, 18, 20, 23]	**Conduct educational meeting s[**12**,** 15**,** 23**]** **Identify and prepare champion s[**15**]** **Capture and share local knowledg e[**15**]** **Conduct local consensus discussion s[**20**,** 23**]** Create a learning collaborative Facilitation Conduct a local needs assessment Assess for readiness and identify barriers and facilitators Recruit, designate and train for leadership Tailor strategies Inform local opinion leaders Promote adaptability Use advisory boards and workgroups	Complexity [18]	Develop a formal implementation blueprint Promote adaptability Conduct small cyclical tests of change Conduct ongoing training Create a learning collaborative Assess for readiness and identify barriers and facilitators Identify and prepare champions Stage implementation scale-up Capture and share local knowledge Model and simulate change Facilitation Identify early adopters Organize clinician implementation team meetings Provide ongoing consultation Tailor strategies
Available resources [18]	**Capture and share local knowledge** **[**18**]** Access new funding Change physical structure and equipment Develop resource sharing agreements Alter patient/consumer fees Fund and contract for clinical innovation Make billing easier Use other payment schemes	Relative advantage [20]	**Conduct local consensus discussions** **[**20**]** Identify and prepare champions Conduct a local needs assessment Conduct small cyclical tests of change Inform local opinion leaders Assess for readiness and identify barriers and facilitators Conduct educational meetings Alter incentive/allowance structures Increase demand Promote adaptability Visit other sites
Engagement [18]	**Conduct local consensus discussions** **[**18**]** Conduct a local needs assessment Assess for readiness and identify barriers and facilitators Develop and implement tools for quality monitoring Identify and prepare champions Develop a formal implementation blueprint Conduct ongoing training Facilitation	Peer pressure [20]	**Conduct local consensus discussions** **[**20**]** **Involve executive boards** **[**20**]** Increase demand Identify early adopters Alter incentive/allowance structures Identify and prepare champions Involve patients/consumers and family members Inform local opinion leaders
Tension for change [10, 14, 15, 17, 18, 21]	**Identify and prepare champions** **[**15**]** **Conduct local consensus discussions** **[**10*,* 17*,* 18**]** **Facilitate relay of clinical data to providers** **[**10*,* 21**]** Involve patients/consumers and family members Inform local opinion leaders Assess for readiness and identify barriers and facilitators Alter incentive/allowance structures Conduct a local needs assessment		

Bold text indicates utilisation in the included studies. The barrier column includes all studies that stated the relevant barrier. Not all barriers reported in the studies were addressed using the recommendations, e.g. complexity

## Results

### Characteristics of eligible studies

Fourteen papers were selected for final inclusion (see Table 1) [10–23]. Study designs included before and after implementation studies (*n* = 3) [10, 20, 23], retrospective observational (*n* = 6) [11–13, 16, 18, 21] and prospective interventional studies (*n* = 5) [14, 15, 17, 19, 22]. The majority of studies were conducted in Europe (*n* = 5) [13, 17, 19, 20, 22] or North America (*n* = 8 )[10–12, 14–16, 18, 23] with one paper from Australia [21]. Half of the included papers studied perioperative patient populations (50%, *n* = 7) [10, 11, 13, 15, 19, 21, 41], while 7% (*n* = 1) were focused on critical care [23], and 43% (*n* = 6) were unspecified [12, 14, 16–18, 20]. Outcome measures/results were reported in multiple formats. The majority (64%, *n* = 9) [10, 12–15, 19, 21–23] reported crude reductions in blood transfusions or 14% (*n* = 2) [20, 23] reported red cell units transfused per patient. The remaining three studies reported red cell units per 1000 patient days [16, 18], and number of patients transfused [17].

### Study quality

Study quality was generally moderate (between 50 and 100%) [40] (Table 1). We did not exclude studies based on quality as this was a descriptive review with no intent for meta-analysis, thus facilitating the investigation of quality issues in the literature. Generally, quality scores were lower due to a failure to provide transparent and detailed demographics, lack of discussion about the measurement instrument or where the designs were uncontrolled. There was also considerable variation in the length of follow up, and in some studies, there was a significant disparity in size between control and intervention groups.

### Barriers

The barriers identified within each paper and the implementation strategies used to address them are summarised in Table 2. Eleven of the 39 CFIR constructs were identified as barriers to implementation including access to knowledge and information (*n* = 7) [10, 12, 13, 16, 18, 21, 22], knowledge and beliefs about the intervention (*n* = 7 )[12, 14–16, 19, 20, 23], tension for change (*n* = 6 )[10, 14, 15, 17, 18, 21], culture (*n* = 4) [12, 15, 20, 23], structural characteristics (*n* = 4) [11, 12, 17, 18], evidence strength and quality (*n* = 3) [12, 16, 20], available resources (*n* = 1) [18], complexity (*n* = 1) [18], engagement (*n* = 1) [18], peer pressure (*n* = 1) [20] and relative advantage (*n* = 1) [20]. Across the papers, a median of three barriers were reported, ranging between one and six.

### Implementation strategies

Twenty-five different implementation strategies were identified in the included studies. The 10 most common implementation strategies were the following: conduct educational meetings (8 studies) [11, 12, 15, 17–19, 21, 23], audit and provide feedback (6 studies) [10–12, 15, 20, 23], develop educational materials (6 studies) [10, 13, 15, 17, 20, 21], conduct local consensus discussions (6 studies) [10, 13, 17, 18, 20, 23], develop and implement tools for quality monitoring (5 studies ) [10, 11, 16, 19, 21], remind clinicians (5 studies) [11, 12, 14, 15, 22], involve executive boards (4 studies) [12, 18, 20, 22], distribute educational materials (3 studies) [16, 20, 22], facilitate relay of clinical data to providers (3 studies) [11, 19, 21], capture and share local knowledge (2 studies) [15, 18]. Across the papers, a median of five implementation strategies were reported, ranging between one and seven.

### ERIC agreement

Table 2 provides a summary of barriers reported and implementation strategies used in each paper and agreement with the ERIC recommendations. Six studies had over 80% agreement, five studies had 50% agreement, one study had 20% agreement, and two studies had no agreement. The median and mode agreement was 50%. Table 3 reports the individual barrier constructs, implementation strategies used to address them and the agreement with the ERIC recommendations. Implementation strategies in italic text indicate utilisation by the relevant study.

## Discussion

This paper is the first to investigate and report barriers to implementation of the PBM guidelines and compare implementation strategies used with those recommended in the ERIC tool (measured as a level of agreement) [33]. Several key findings (in the context of PBM guidelines) became evident during the review. Firstly, only one paper reported the use of an implementation strategy, demonstrating poor knowledge, understanding and application of implementation theory and frameworks in general. Secondly, the reporting of implementation studies is weak and requires improvement. While there was a cluster of barriers that were common amongst studies, there was high heterogeneity in the implementation strategies used. Finally, the ERIC tool can be used to provide guidance but requires further work to ascertain a strong consensus for recommended implementation strategies across all barriers.

Despite the existence of multiple theories and frameworks to help guide barrier identification, intervention selection and implementation process, only one study referred to a formal implementation theory, model or framework or existing quality improvement methods, although the authors did not explicitly state what it was [15]. This problem is not unique to PBM, and a recent review by Wensing and Grohl highlights the lack of theoretically informed implementation as a wider issue within implementation science [27]. Part of the problem may be the sheer number of tools available, and also, the knowledge required to identify and apply them appropriately [26]. As a result, the literature provides generalised reports about the effect of implementation strategies in local settings and fails to explain and report any implementation preparation undertaken or provide explicit detail as to the context in which the implementation occurred [12–14, 16–21, 23, 41]. The absence of a reported methodological approach presents a missed opportunity to test the effectiveness of implementation attempts rigorously. Future research should utilise available implementation methodologies to help improve the understanding of how to translate evidence to practice.

The quality of reporting of included studies was generally low and supports observations made by Luoto and colleagues that standards to help improve reporting quality in implementation studies are needed [42]. Standards that provide guidance are available, for example, the Standards for Reporting Implementation Studies (StaRI) [43], and they should be used routinely. The StaRI guidelines provide recommendations for both reporting an intervention and the associated implementation strategy [43]. The utilisation of the standards and improved reporting will provide the foundations for the validation and advancement of implementation theory, both in terms of describing interventions and the strategies used to implement them [42, 43]. All articles included focused on reporting the impact of implementation strategies used, and only one reported on an implementation model or theory but explained it only as having used “standard quality improvement methodologies” [15]. Just over half of the included studies attempted to tailor intervention strategies to their relevant context using local consensus processes [10, 13, 17, 18, 20, 23]. It is advisable to ensure that implementation strategies are tailored to ensure compatibility with existing processes and acceptability of staff. A relevant example in the context of PBM is using the strategy of audit and feedback to help clinicians identify opportunities for improving practice. As recently outlined in a systematic review by Brown and colleagues, tailoring audits to local context is crucial to ensure the success of audit and feedback [44].

There was a cluster of common barriers reported in the included studies. However, in-depth explanation and exploration of barriers were limited in most articles, perhaps as the focus of the research was on describing the implementation strategies and how well they worked (i.e. reduction in red blood cell utilisation). The most common barriers reported in the studies were knowledge and beliefs about the intervention, access to knowledge and information, and tension for change. Knowledge and beliefs about the intervention was a barrier in seven papers and is defined as the individual’s attitude and a general understanding of the key principles of an intervention [33]. Many papers acknowledge that this was a barrier for their facility and used various implementation strategies to educate their staff about their local PBM guidelines and why they are essential for patient safety. Interventions to support the implementation strategies included distributing information (e.g. pamphlets) [16, 22], setting up online learning portals [16], holding educational sessions at grand rounds [11], and implementation of performance tracking dashboards [45, 46]. ERIC strategies that were not used to address this barrier included identifying local barriers, conducting a needs assessment and informing local opinion leaders. The utilisation of these implementation strategies to address the knowledge and belief barrier may enhance implementation efforts [33].

Access to knowledge and information was a barrier in seven papers and is defined as the availability of resources that provide education and guidance to support the uptake of an intervention [29]. Reporting of access to knowledge and information as a barrier included the acknowledgement of the absence of a contemporary local protocol or policy to guide transfusion decision-making [13]. The development of policy and procedure using local consensus discussions was undertaken in some instances [15]. The changes were then disseminated through educational meetings [12, 18, 21]. ERIC strategies that were not used to address this barrier centred around pragmatic educational implementation strategies, including conducting educational outreach visits, providing technical assistance and shadowing experts. The utilisation of these implementation strategies to address the access to knowledge and information barrier may enhance implementation efforts [33].

The tension for change (or rather, absence of) was a barrier in six papers, and this refers to the degree to which stakeholders perceive that change as necessary [29]. Reporting of tension for change included identified variability in practice, ignorance of best practice guidelines and current hospital performance [10, 14, 15, 17, 18, 21]. The variability and lack of awareness was compounded by outdated practices, proliferated through myths held by some senior physicians, (for example, the dictum “if you are going to transfuse, you might as well use two units”), based on the premise that one unit was never adequate [12, 16]. Audit and feedback [10, 15] were utilised to provide clinicians with insight into their practice as well as the conduct of educational meetings [15, 17, 18, 21] to educate clinical staff on what is considered best practice. ERIC strategies that were unused included involving consumers and family, conducting local needs assessments, informing local opinion leaders, assessing barriers and altering incentive structures. The utilisation of these implementation strategies to address the tension for change barriers may enhance implementation efforts.

There was a high variation in the implementation strategies used in the included studies, with 25 different implementation strategies employed across the papers. Recent research undertaken by Althoff and colleagues included a meta-analysis of the effect of multimodal patient blood management programs and noted high heterogeneity of implementation strategies, supporting this finding [8]. Their review analysed implementation strategies used and their impact on red blood cell transfusion reduction but did not seek to understand the barriers faced by health professionals [8]. Health professionals would benefit from more explicit guidance as to which implementation strategies would best suit their local context. In order to use the data summarised in this paper, health professionals should use an implementation model or framework (e.g. CFIR) to help identify local barriers to see what has worked before, in the context of what is recommended by the ERIC tool [33]. Future research should focus on testing well-described implementation strategies, tailored to the local context.

The authors of the ERIC tool have commented that there was surprising heterogeneity between consensus for implementation strategies and acknowledge that further work is required to advance the utility of the tool [33]. The ERIC tool provided recommendations for ten of the barriers that were present in the included study [33]. One barrier had no moderate or strong recommendations, which was complexity, although this construct was only identified in one paper. Further refinement of the tool and the conduct of PBM implementation studies that utilise rigorous implementation science methodologies such as the ERIC tool, with quality reporting processes are needed to provide further guidance.

This review has several important limitations, the first of which is that many of the included papers were not written with the intent of reporting or analysing local barriers and implementation strategies that were used to address them. A large number of papers (*n* = 62) that would have been useful in terms of understanding the implementation strategies used and their impact on practice improvement were excluded because they did not explicitly mention existing barriers. The final limitation is the quality of reporting of interventions and implementation strategies used in the papers, which was generally quite poor. It is difficult to know if every implementation strategy and every intervention was mentioned in the papers, and this may have impacted on the ERIC agreement. We also acknowledge that the restricted review method chosen has potential limitations as we did not search for grey literature and the use of pilot sampling during screening means that we cannot be certain that all relevant literature was included.

## Conclusion

The results of this review identified a cluster of barriers within PBM guideline implementation that consisted of 11 of 39 CFIR constructs. Despite the common barriers, there was high heterogeneity in the implementation strategies used by health professionals, with over 25 utilised. The most common barriers reported in the studies were knowledge and beliefs about the intervention, access to knowledge and information and tension for change. Common implementation strategies selected to address the barriers included conducting educational meetings, auditing and providing feedback, the development of educational materials, and conducting local consensus discussion. Health professionals should find these implementation strategies useful for addressing barriers to evidence-based patient blood management practice. Only one paper provided an explicit reference to having used an implementation model or framework, but it appears that in many (not all) instances, included papers were able to identify, and subsequently address most barriers, with the majority of studies demonstrating strong agreement with the ERIC tool. The utilisation of implementation frameworks and complementary tools may have enhanced this process. Studies need to utilise and report on implementation frameworks and tools to advance the field. Further refinement of the ERIC tool to include strong recommendations for all barriers would be advantageous in assisting health care professionals in selecting appropriate implementation strategies.

## Data Availability

All data generated or analysed during this study are included in this published article.

## Abbreviations

CFIR: Consolidated Framework for Implementation Research
ERIC: Expert Recommendations for Implementing Change
PBM: Patient blood management
PRISMA: Preferred reporting items for systematic review and meta-analysis protocols)
StaRI: Standards for Reporting Implementation studies
